# The effectiveness of ultrasound-guided core needle biopsy in detecting lymph node metastases in the axilla in patients with breast cancer: systematic review and meta-analysis

**DOI:** 10.1016/j.clinsp.2023.100207

**Published:** 2023-05-02

**Authors:** Qi Xu, Jiale Wang, Jing Wang, Runzhao Guo, Yao Qian, Feng Liu

**Affiliations:** a Department of Breast Surgery, Harbin Medical University Cancer Hospital, Harbin, China; b Department of Medical Oncology, Harbin Medical University Cancer Hospital, Harbin, China

**Keywords:** Core needle biopsy, Breast cancer, Meta-analysis, Diagnostic, Axillary lymph nodes

## Abstract

**Objective:**

This study aimed to perform a meta-analysis to investigate the diagnostic safety and accuracy of Ultrasound-Guided Core Needle Biopsy (US-CNB) Axillary Lymph Nodes (ALNs) region in patients with Breast Cancer (BC).

**Methods:**

The authors searched the electronic databases PubMed, Scopus, Embase, and Web of Science for clinical trials about US-CNB for the detection of ALNs in breast cancer patients. The authors extracted and pooled raw data from the included studies and performed statistical analyses using Meta-DiSc 1.4 and Review Manager 5.3 software. A random effects model was used to calculate the data. At the same time, data from the Ultrasound-guided Fine-Needle Aspiration (US-FNA) were introduced for comparison with the US-CNB. In addition, the subgroup was performed to explore the causes of heterogeneity. (PROSPERO ID: CRD42022369491).

**Results:**

In total, 18 articles with 2521 patients were assessed as meeting the study criteria. The overall sensitivity was 0.90 (95% CI [Confidence Interval], 0.87‒0.91; *p* = 0.00), the overall specificity was 0.99 (95% CI 0.98‒1.00; *p* = 0.62), the overall area under the curve (AUC) was 0.98. Next, in the comparison of US-CNB and US-FNA, US-CNB is better than US-FNA in the diagnosis of ALNs metastases. The sensitivity was 0.88 (95% CI 0.84‒0.91; *p* = 0.12) vs. 0.73 (95% CI 0.69‒0.76; *p* = 0.91), the specificity was 1.00 (95% CI 0.99‒1.00; *p* = 1.00) vs. 0.99 (95% CI 0.67‒0.74; *p* = 0.92), and the AUC was 0.99 vs. 0.98. Subgroup analysis showed that heterogeneity may be related to preoperative Neoadjuvant Chemotherapy (NAC) treatment, region, size of tumor diameter, and the number of punctures.

**Conclusion:**

US-CNB has a satisfactory diagnostic performance with good specificity and sensitivity in the preoperative diagnosis of ALNs in BC patients.

## Highlights

• US-CNB is a reliable preoperative test for detecting axillary metastasis in breast cancer patients, with high accuracy and safety.
• Compared to US-FNA, US-CNB shows even higher accuracy for detecting axillary metastasis in breast cancer patients.
• The use of US-CNB as a preoperative test can reduce the need for a second operation and improve patient outcomes.

## Introduction

Breast Cancer (BC) is now one of the most common malignancies in women worldwide. It has a high morbidity and mortality rate and is one of the leading causes of death in women, posing a major threat and challenge to women's health worldwide.^1^ Preoperative assessment of Axillary Lymph Nodes (ALNs) in BC patients allows early and accurate staging of patients and plays an important role in the subsequent treatment and prognostic assessment and can be used to make relevant decisions about adjuvant therapy such as radiation treatment, Neoadjuvant Chemotherapy (NAC) treatment, and breast reconstruction. According to the results of the American College of Surgeons Oncology Group (ACOSOG) Z0011 trial, patients with breast-conserving early-stage BC with tumors less than 5 cm can safely avoid Axillary Lymph Node Dissection (ALND) even if SLN1-2 metastases are treated with subsequent breast radiotherapy and systemic therapy.^2^ The release of the Z0011 trial, which changed the traditional changed the traditional treatment approach of the past and set off an intense debate and clinical practice.^3^ Among them, ALND impacts patients' overall postoperative quality of life and can be associated with significant surgical complications (pain, upper extremity mobility impairment, edema, and so on).^4^ Ultrasound (US) is often an important tool in the preoperative diagnosis of morphological features of ALNs and is key to understanding the progression of BC. With the increasing availability of ultrasound, the accuracy of preoperative diagnosis in BC patients is improving.

Ultrasound-guided Fine Needle Aspiration (US-FNA) and ultrasound-guided core needle biopsy (US-CNB) use ultrasound imaging to select the optimal puncture biopsy route, effectively improving the accuracy of diagnosis. In recent years, the use of US-FNA for preoperative evaluation of ALNs has increased due to its low risk, ease of use, low cost, and low complication rate.^5^ Recently, it has been suggested that US-CNB may be superior to US-FNA in terms of diagnostic accuracy, providing a more accurate preoperative assessment of the status of ALNs and potentially replacing US-FNA.^6^

US-CNB is a technique in which ALNs tissue is removed from an abnormal area of the axilla, usually by an operator using a large core needle from an ultrasonically explored area.^7^ Besides having good accuracy, the US-CNB offers more extra samples for tumor classification and immunohistochemistry.^8^ But it is more expensive and more invasive, and there may be a possible danger of malignant seeding along the puncture path.

The diagnostic value of the US-CNB has been questioned due to the lack of consistency in the various reported results. There have been previous studies on the accuracy of US-CNB for the axilla, but fewer studies have been included. Based on the above, the authors present an updated review and meta-analysis. Thus, the authors have mainly comprehensively assessed the diagnostic performance of US-CNB in assessing metastases of ALNs in BC to provide a basis for accurate preoperative assessment.

## Methods

The review complies with the Preferred Reporting Items for Systematic Reviews and Meta-Analyses (PRISMA).^9^ And the review complied with the code of the “Cochrane Handbook for Systematic Reviews of Diagnostic Test Accuracy”.^10^ The protocol was registered and accepted by PROSPERO under CRD42022369491.

### Literature search

The authors searched the electronic databases PubMed, Scopus, Embase, and Web of Science for clinical trials about US-CNB for the detection of ALNs in breast cancer patients. The following search terms were used: ((sonography OR sonography guided) OR (Ultrasound guided OR US-guided) OR (Ultrasound OR US)) AND (core-needle biopsy OR core needle biopsy OR CNB) AND (ALN OR axillary lymph nodes OR axillary lymphadenopathy OR axillary staging) AND (pre-operative staging OR preoperative OR preoperative period OR preoperative). The start date of the search was not restricted. Our last search was updated on 1 September 2022. Among them, search results were combined and output to the EndNote bibliographic management tool, and repeated results were deleted.^11^ Next, full-text screening was performed to include eligible ones in the meta-analysis. The authors conducted a manual search of the literature included in the study. Eligibility screening of the literature included was carried out independently by two trained researchers. Any disagreements were resolved by consensus or by consultation with a third senior researcher. In addition, the screening process of the study was summarized by the PRISMA flowchart.

### Inclusion and exclusion criteria

Two researchers independently reviewed the titles, abstracts, and full text of the original articles. Two researchers screened eligible articles for inclusion and included those that met the following criteria: 1) BC Patients with no preoperative clinical signs of ALNs metastasis; 2) Included studies should have enough data; 3) Published case-controlled study of the accuracy of the US-CNB correlation; 4) Retrospective and prospective studies. Next, the authors further reviewed the articles and excluded those that met the exclusion criteria, and the excluded articles comply with the below criteria: 1) Articles that have been repeatedly published; 2) Literature with unavailable full papers or insufficient data; 3) Abstracts, conference articles, case reports, dissertations, and so on; 4) Animal laboratory experiments; 5) Non-English language articles and; 6) Pregnancy studies.

### Data extraction

All study data in this review were collected independently by two researchers. The following data were collected: the first author, study type, number of patients, country, mean age of patients, needle diameter, number of tTrue Positives (TP), number of False Positives (FP), number of False Negatives (FN), and number of True Negatives (TN). In the process of original data collection, some studies performed US-CNB and US-FNA at the same time, and the authors collected both together for subsequent comparative analysis.

### Quality assessment

The quality of the included literature was independently assessed using the Cochrane Handbook diagnostic study quality assessment tool QUADAS-2, which was used for this diagnostic accuracy meta-analysis to determine the level of risk bias and applicability for each of these parameters.^12^ It consists of four key areas (patient selection, index test, reference standard, and flow and time) to help assess the risk of bias. There are three levels of risk of bias based on the likelihood of bias occurring: high risk, unclear risk, and low risk. The quality assessment is carried out by two researchers after reading the full text, and if they cannot reach a consensus, a third senior researcher should be consulted to resolve the issue.

### Statistical analysis

Statistical analysis of the data collected and adherence to the Cochrane Diagnostic Test Accuracy (DTA) review guidelines.^10^ Meta-analysis of the included literature data was carried out using Meta-DiSc1.4 software and Review Manager 5.3 software, and heterogeneity was evaluated by I^2^ statistics.

During the statistical analysis of diagnostic studies, it is inevitable to encounter heterogeneity, which may be caused by threshold effects or non-threshold effects. Heterogeneity due to threshold effects was tested using the Spearman correlation coefficient. If p > 0.1, it indicates that the threshold effect did not cause heterogeneity; if p≤0.1, it indicates that the threshold effect caused heterogeneity. Heterogeneity was evaluated using the I^2^ statistics, with scores from 25%–49% classified as low, 50%–74% as moderate, and more than 75% as high heterogeneity.^13^ Meanwhile, the authors plotted the Summary Receiver Operating Characteristic(SROC) curve of US-CNB to illustrate the association for sensitivity and specificity, where the value of AUC is 0.5 and 0.7 means poor precision, > 0.7 and 0.9 means middle precision, > 0.9 and 1.0 means great precision. Meta-analysis results are mainly presented in the form of forest plots, and funnel plots are used to analyze for publication bias.

## Results

### Search results

**Fig. 1:**
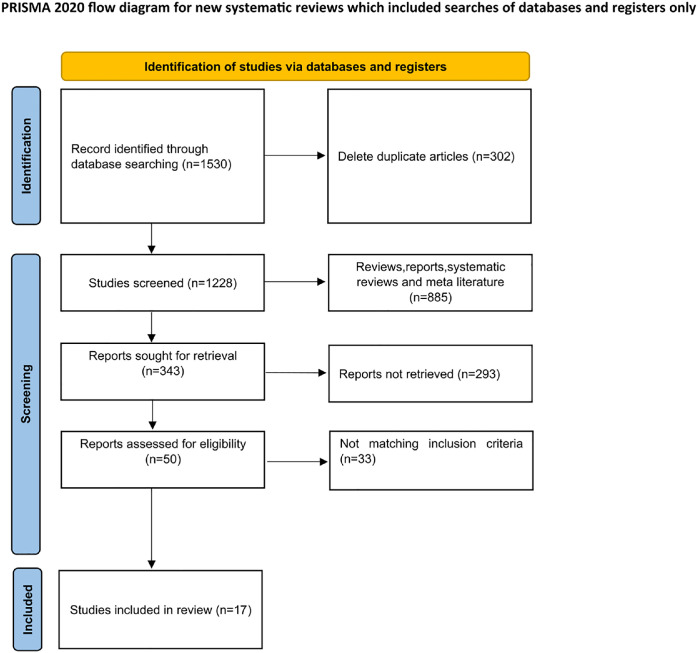
PRISMA flow chart summarizing the process of selecting included studies.

The authors tentatively identified 1530 studies in the literature search, and after excluding duplicates, a total of 1228 studies remained. 343 studies remained after the exclusion of reviews, reports, system reviews, and meta-analyses. 343 articles were assessed from the full text and 17 studies were finally included for meta-analysis. Details of the process used to select the included studies are shown in Fig. 1.

### Characteristics of included studies

Of the 17 studies, 7 were retrospective and 10 were prospective. Of these, eight studies compared US-CNB with US-FNA. Regionally, there were five studies in Asia, seven studies in Europe, and five studies in the United States. The needle diameters used in US-CNB varied between 14- and 22-gauge, with eight studies using 14-gauge needles and others using 16-, 18-, 20-, 21-, and 22-gauge needles or not reporting. Needle diameters used in US-FNA varied between 21 and 25 gauges, with six studies using 21-gauge and 25-gauge needles. Of these, most patients had invasive tumors (Table 1).

**Table 1:** Characteristics of US-CNB patients and studies included in the meta-analysis.

Author	Year	Type of study	Country	Age	Patients	TP	TN	FP	FN	Needle-diameter (g)
Rao et al.^14^	2009	Retrospective	USA	52.5	25	18	3	0	4	18
Topal et al.^15^	2005	Prospective	Turkey	51	39	30	6	0	3	16
Nori et al.^16^	2007	Prospective	Italy	56.4	14	11	2	0	1	14‒16
Hu et al.^17^	2021	Prospective	China	51	167	139	20	0	8	22
Riedel et al.^18^	2021	Retrospective	Germany	56	64	35	20	0	9	‒
Bhandari et al.^19^	2018	Prospective	China	53.1	131	45	81	0	5	16
Rautiainen et al.^20^	2013	Prospective	Finland	61.4	66	45	15	0	6	20
Topps et al.^21^	2018	Retrospective	UK	55.1	92	46	37	0	9	14
Nakamura et al.^22^	2018	Prospective	Japan	56	272	100	160	0	12	21
Ahn et al.^23^	2013	Prospective	Korea	49	48	20	22	0	6	16‒18
Hackney et al.^24^	2013	Prospective	UK	65	46	24	14	0	8	14
Nathanson et al.^25^	2007	Prospective	USA	59.3	121	52	55	3	9	14‒18
Solon et al.^26^	2012	Retrospective	Irish	-	121	110	7	0	4	18‒20
Abe et al.^27^	2009	Retrospective	USA	55	100	64	32	0	4	14
Vidya et al.^6^	2017	Prospective	UK	-	38	27	11	0	0	14
Ganott et al.^28^	2014	Prospective	USA	-	95	61	25	0	9	14
Henry-Tillman et al.^29^	2015	Retrospective	USA	53	58	45	8	0	5	14

TP, True Positive; TN, True Negative; FP, False Positive; FN, False Negative; US-CNB, Ultrasound-guided Core Needle Biopsy.

### Quality and risk of bias

**Fig. 2:**
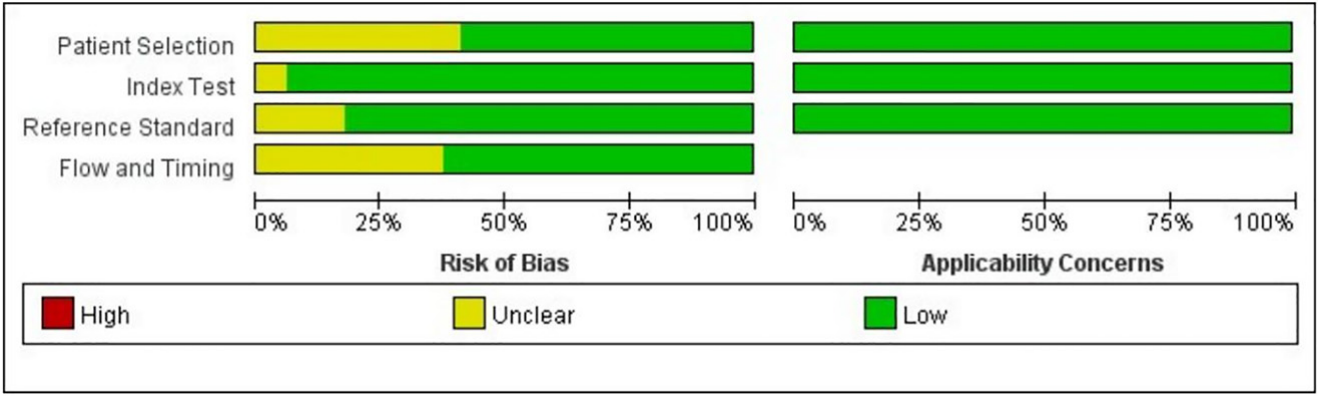
Graph of the risk of bias for the included literature.

**Fig. 3:**
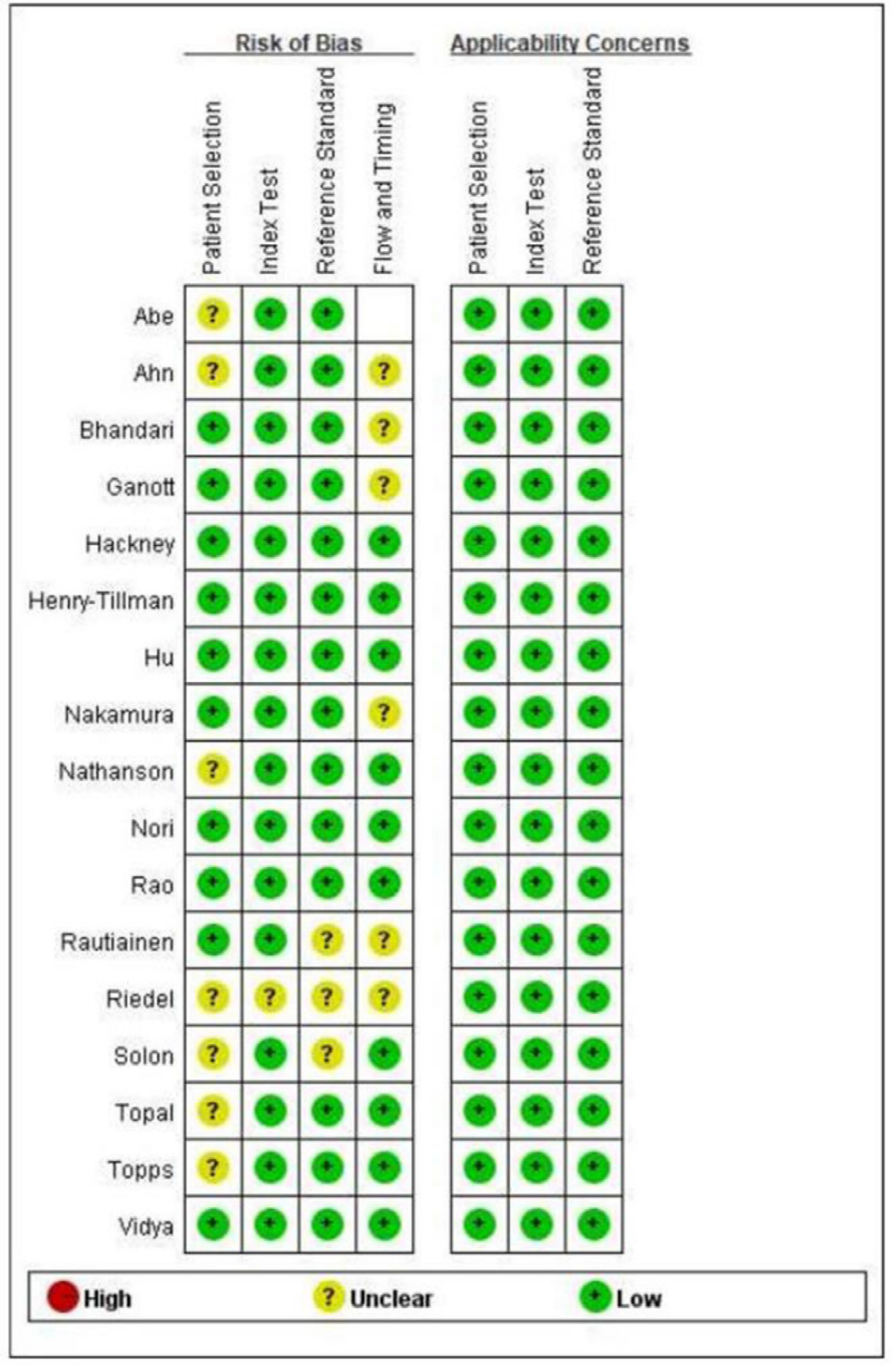
Summary graph showing the risk of bias assessed for the included literature.

**Fig. 4:**
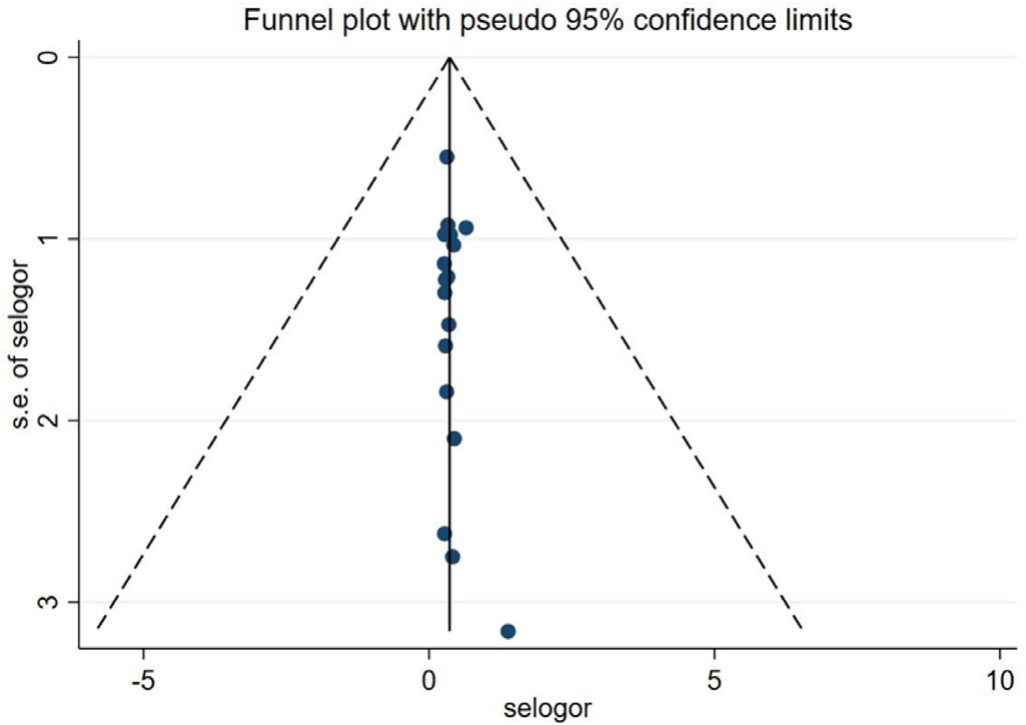
Funnel plot of publication bias.

Because a portion of the study selection contained neoadjuvant patients and a portion of patients was not formally randomized to the US-FNA or US-CNB groups for the trial, there is some risk of uncertainty in the study, resulting in approximately half of the uncertainty in patient selection, flow, and timing. In index text and flow and timing, most studies used standardized postoperative pathology findings as a criterion, showing a low risk of bias. Fig. 2, Fig. 3 show the overall risk of bias in the results for every study and the pooled results, and the overall quality is quite good. The 17 circles in the US-CNB publication bias funnel plot indicate the 17 studies in the review, and the middle line indicates the total sum of DOR. The distribution of the 17 circles showed a great deal of symmetry, indicating no significant publication bias (Fig. 4).

### Diagnostic accuracy of US-CNB for diagnosing ALNs

**Fig. 5:**
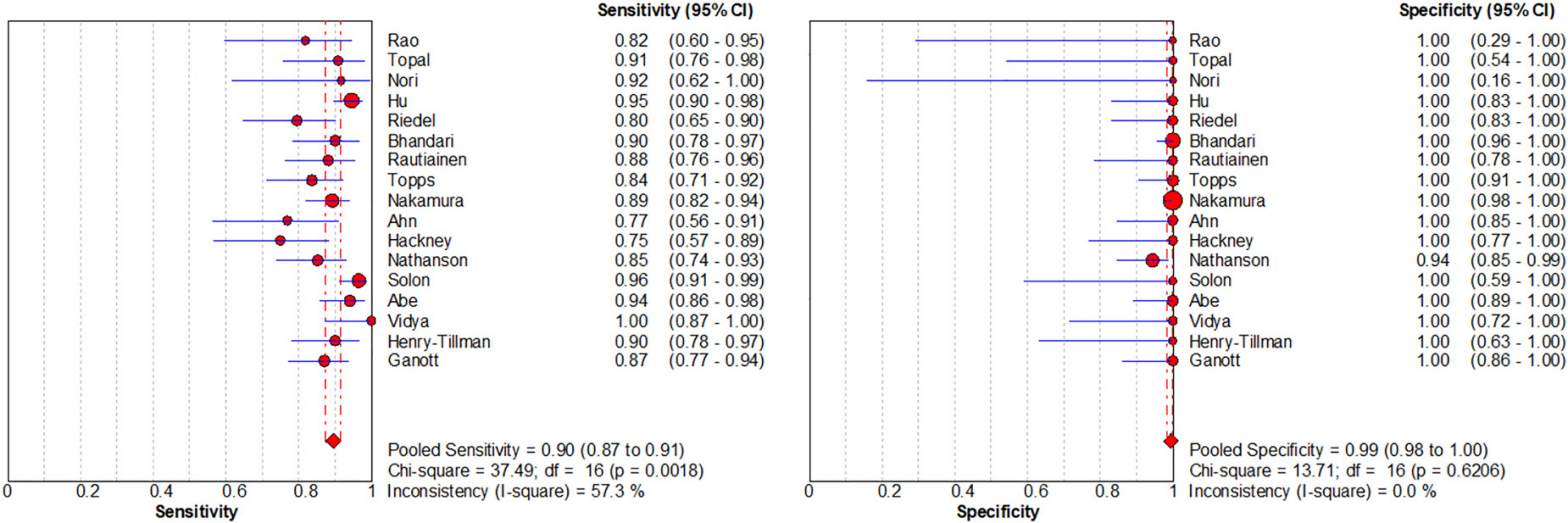
Forest plots of sensitivity (left) and specificity (right) for US-CNB.

**Fig. 6:**
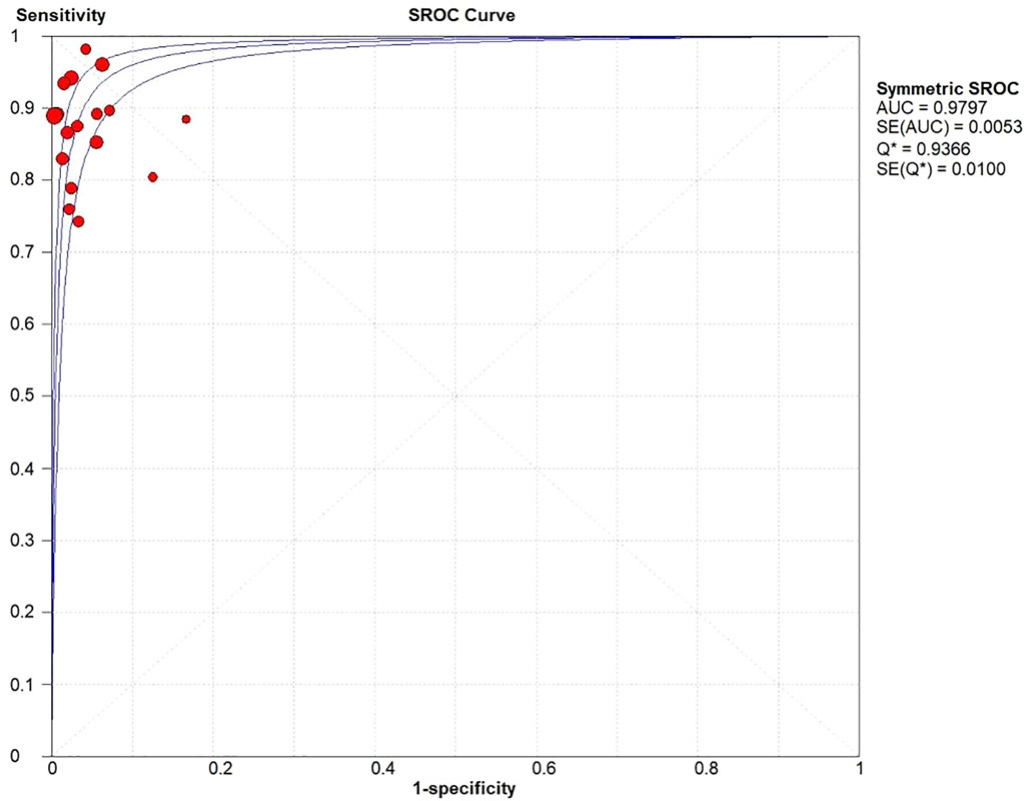
The SROC curve for US-CNB.

The authors plotted the forest plot of the specificity and sensitivity of US-CNB in the diagnosis of ALNs metastasis (Fig. 5). The overall sensitivity of the US-CNB for the detection of ALNs metastases from BC was 0.90 (95% CI [confidence interval] 0.87‒0.91; *p* = 0.00). Pooled studies were heterogenous (I*^2^* = 57.30%). The overall specificity of the US-CNB for the detection of ALNs metastases from BC was 0.99 (95% CI 0.98‒1.00; *p* = 0.62). Pooled studies were homogenous (I*^2^* = 0.00%). To assess the diagnostic value and predictive accuracy of the US-CNB, it was fully evaluated using SROC, which had an Area Under the Curve (AUC) of 0.9797 (Fig. 6). The closer the AUC is to 1.0, the higher the performance of the diagnosis. These supported a good ability of US-CNB to distinguish ALNs metastases from BC.

### Comparison of the accuracy between US-CNB and US-FNA

**Fig. 7:**
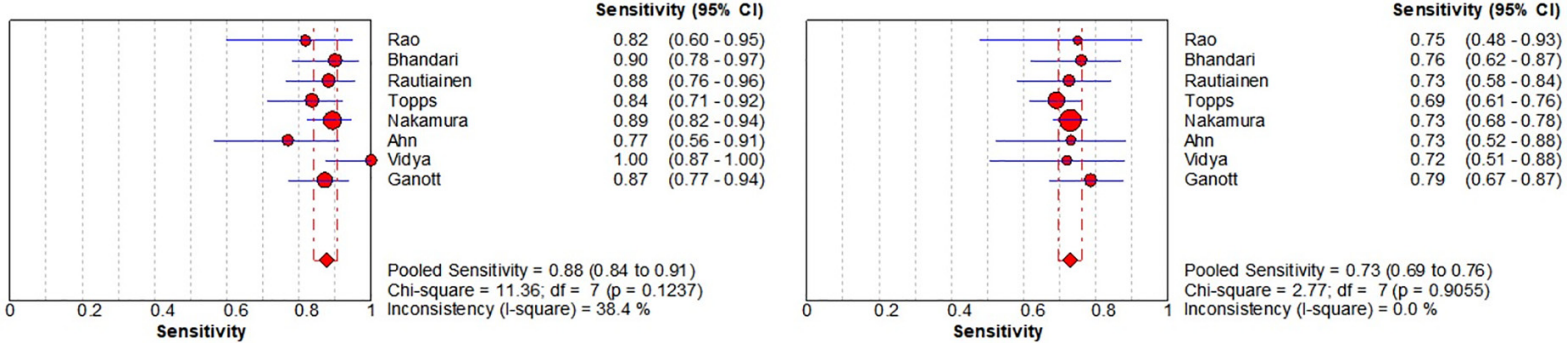
Forest plots of sensitivity of US-CNB (left) and US-FNA (right).

**Fig. 8:**
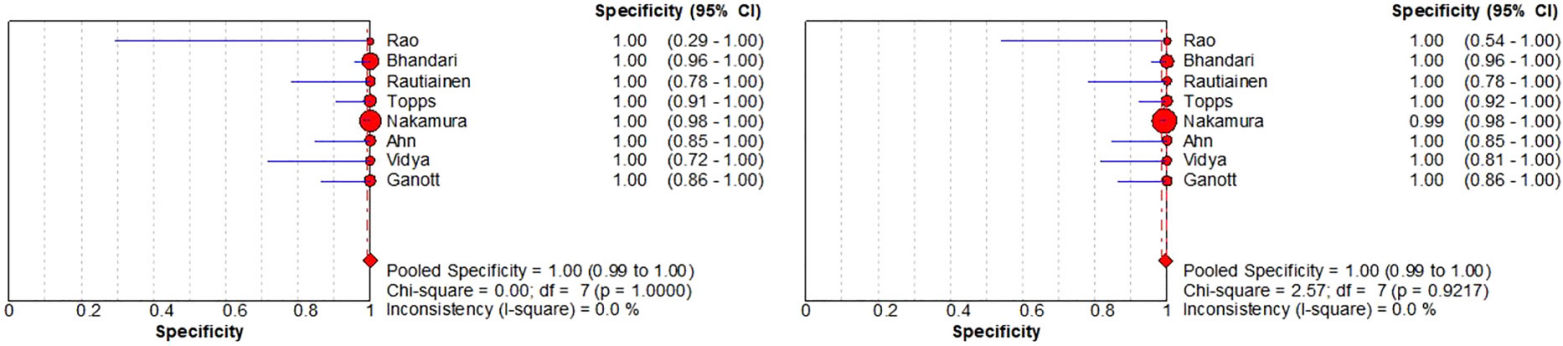
Forest plots of specificity of US-CNB (left) and US-FNA (right).

**Fig. 9:**
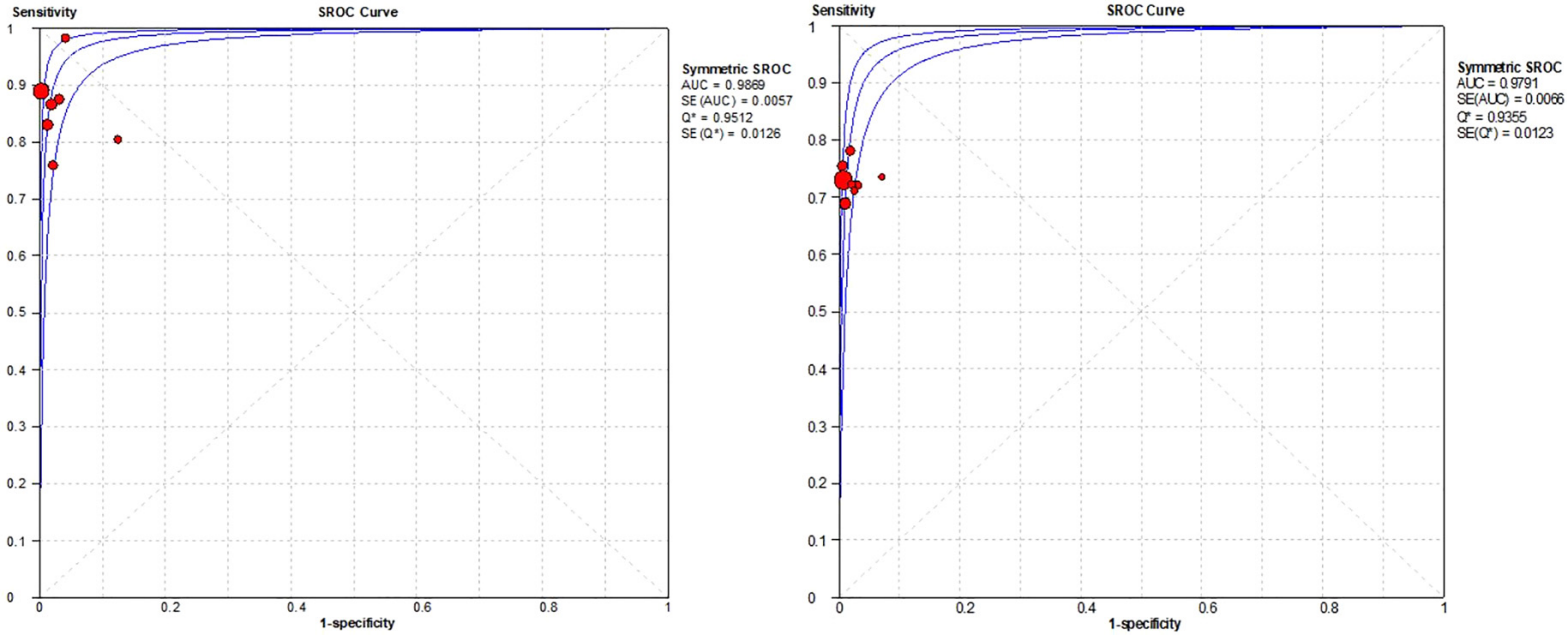
The SROC curve for US-CNB (left) and US-FNA (right).

Nine of the articles in our review include comparative studies of US-CNB and US-FNA (Table 2). Next, the authors compare the diagnostic value of US-CNB and US-FNA by summarizing these 9 articles. As shown in Fig. 7, in terms of overall sensitivity for the detection of ALNs metastases from BC, US-CNB was 0.88 (95% CI 0.84‒0.91; *p* = 0.12) vs. US-FNA was 0.73 (95% CI 0.69‒0.76; *p* = 0.91). The pooled studies of US-CNB were heterogeneous (I^2^ = 38.40%) and of US-FNA were homogenous (I^2^ = 0.00%). At the same time in Fig. 8, in terms of overall specificity for the detection of ALNs metastases from BC, US-CNB was 1.00 (95% CI 0.99‒1.00; *p* = 1.00) vs. US-FNA was 1.00 (95% CI 0.99‒1.00; *p* = 0.92). The pooled studies of US-CNB and US-FNA were both homogenous (I^2^ = 0.00%). Then, the authors plotted the SROC curves for US-CNB and US-FNA, which yielded an AUC of 0.99 vs. 0.98 in Fig. 9. By comparing their sensitivity, specificity, and SROC curves, the authors found that US-CNB was slightly better than US-FNA in detecting ALNs metastases from BC.

**Table 2:** Summary of studies containing US-CNB and US-FNA comparisons.

Author	US-CNB						US-FNA					
	Patients	TP	TN	FP	FN	Needle-diameter (g)	Patients	TP	TN	FP	FN	Needle-diameter (g)
Rao et al.^14^	25	18	3	0	4	18	22	12	6	0	4	25
Bhandari et al.^19^	131	45	81	0	5	16	131	38	81	0	12	21
Rautiainen et al.^20^	66	45	15	0	6	20	66	37	15	0	14	21‒22
Topps et al.^21^	92	46	37	0	9	14	215	118	44	0	53	21
Nakamura et al.^22^	272	100	160	0	12	21	744	254	393	3	94	21
Ahn et al.^23^	48	20	22	0	6	16‒18	48	19	22	0	7	21
Vidya et al.^6^	38	27	11	0	0	14	43	18	18	0	7	21
Ganott et al.^28^	95	61	25	0	9	14	95	55	25	0	15	21‒25

TP, True Positive; TN, True Negative; FP, False Positive; FN, False Negative; US-CNB, Ultrasound-guided Core Needle Biopsy; US-FNA, Ultrasound-guided Fine-Needle Aspiration.

### Heterogeneity

The I^2^ value of the overall sensitivity of the US-CNB was > 50%, indicating a moderate degree of heterogeneity. Spearman correlation analysis was used to further investigate the source of heterogeneity. The results showed *r* = 0.080, *p* = 0.759, not statistically significant, and no correlation. The results suggest that the moderate heterogeneity in the overall sensitivity of the US-CNB is independent of the threshold effect. The heterogeneity may be due to the remaining causes. The authors then performed subgroup analyses to further explore the sources of heterogeneity.

### Subgroup analysis

The use of preoperative NAC treatment before US-CNB testing may lead to the misclassification of tumors as negative, with implications for the accuracy of ALNs detection.^30^ First of all, the authors divided the overall into two groups (preoperative treatment with NAC and preoperative treatment without NAC) for subgroup analysis, and the results are shown in Table 3. In terms of sensitivity, the use of preoperative NAC treatment was 0.89 (95% CI 0.86‒0.92; *p* = 0.00) versus no usag of preoperative NAC treatment was 0.90 (95% CI 0.87‒0.92; *p* = 0.03). The pooled studies were both heterogenous (I^2^ = 69.30% and I^2^ = 48.40%). In terms of specificity, the use of preoperative NAC treatment was 0.98 (95% CI 0.95‒1.00; *p* = 0.29) versus no usag of preoperative NAC treatment was 1.00 (95% CI 0.99‒1.00; *p* = 1.00). The pooled studies of the use of preoperative NAC treatment were heterogeneous (I^2^ = 17.50%) and those of no use of preoperative NAC treatment were homogenous (I^2^ = 0.00%).

**Table 3:** Subgroup analysis based on NAC for accuracy of US-CNB.

	Category	Number of studies	Sensitivity (95% CI) (p-value), (I^2^%)	Specificity (95% CI) (p-value), (I^2^%)
**NAC**	Use	7	0.89 (0.86‒0.92) (*p* = 0.00) (69.3)	0.98 (0.95‒1.00) (*p* = 0.29) (17.5)
	No-use	11	0.90(0.87‒0.92) (*p* = 0.03) (48.4)	1.00 (0.99‒1.00) (*p* = 1.00) (0.0)
**Pooled estimate**		18	0.90(0.87‒0.91) (*p* = 0.00) (57.3)	0.99 (0.98‒1.00) (*p* = 0.62) (0.0)

95% CI, 95% Confidence Intervals; US-CNB, Ultrasound-guided Core Needle Biopsy; NAC, Neoadjuvant Chemotherapy.

Secondly, the authors decided to divide all the studies into three groups (Asia, Europe, and the United States) by region. The results show that the accuracy varied by region. As shown in Table 4, in three groups, sensitivity and specificity were better in Asia than in Europe and the USA, possibly due to the large number of patients studied in the Asian region.

**Table 4:** Subgroup analysis based on a region for accuracy of US-CNB.

	Category	Number of Patients	Sensitivity (95% CI) (p-value), (I^2^%)	Specificity (95% CI) (p-value), (I^2^%)
**Region**	Asia	657 (44%)	0.91 (0.87‒0.94) (*p* = 0.10) (47.4)	1.00(0.99‒1.00) (*p* = 1.00) (0.0)
	Europe	441 (29%)	0.89 (0.85‒0.92) (*p* = 0.00) (75.7)	1.00(0.97‒1.00) (*p* = 1.0) (0.0)
	United States	399 (27%)	0.89 (0.84‒0.92) (*p* = 0.38) (4.3)	0.98(0.93‒0.99) (*p* = 0.29) (19.8)
**Pooled estimate**		1497(100%)	0.90 (0.87‒0.91) (*p* = 0.00) (57.3)	0.99 (0.98‒1.00) (*p* = 0.62) (0.0)

95% CI, 95% Confidence Intervals; US-CNB, Ultrasound-guided Core Needle Biopsy.

Usually, the larger the BC volume, the faster the proliferation and the longer the growth time of the tumor cells, as well as the higher the likelihood of metastasis of ALNs. And then, the authors decided to divide all studies into two groups (< 2.5 cm and > 2.5 cm) according to the clinical tumor size of the breast cancer. The results are shown in Table 5. Sensitivity and specificity increased with increasing clinical tumor size.

**Table 5:** Subgroup analysis based on clinical tumor size for accuracy of US-CNB.

	**Category**	**Number of studies**	**Sensitivity (95% CI) (p-value), (I^2^%)**	**Specificity (95% CI) (p-value), (I^2^%)**
**Clinical tumor size**	< 2.5cm	7	0.88 (0.84‒0.91) (*p* = 0.72) (0.0)	0.99 (0.95‒1.00) (*p* = 0.08) (46.3)
	> 2.5cm	6	0.91 (0.87‒0.93) (*p* = 0.09) (46.8)	1.00 (0.97‒1.00) (*p* = 1.00) (0.0)
**Pooled estimate**		13	0.89 (0.87‒0.91) (*p* = 0.27) (17.5)	0.99 (0.98‒1.00) (*p* = 0.36) (8.2)

95% CI, 95% Confidence Intervals; US-CNB, Ultrasound-guided Core Needle Biopsy.

Finally, the authors analyzed whether the number of punctures could have an impact on the diagnostic accuracy of US-CNB. The authors divided all studies into two groups (< 3 and > 3) by the number of punctures. The authors found that sensitivity increased with the number of punctures, but specificity did not improve, which may be related to insufficient sample size (Table 6). Overall, the use of preoperative NAC treatment, different regions, clinical tumor size, and the number of punctures may be factors affecting the heterogeneity.

**Table 6:** Subgroup analysis based on number of punctures for accuracy of US-CNB.

	Category	Number of studies	Sensitivity (95% CI) (p-value), (I^2^%)	Specificity (95% CI) (p-value), (I^2^%)
Number of punctures	< 3	10	0.88 (0.85‒0.90) (*p* = 0.27) (18.7)	1.00 (0.99‒1.00) (*p* = 1.00) (0.0)
	> 3	4	0.91 (0.87‒0.94) (*p* = 0.09) (53.9)	0.96 (0.90‒0.99) (*p* = 0.45) (0.0)
Pooled estimate		14	0.89 (0.86‒0.91) (*p* = 0.11) (33.2)	0.99 (0.98‒1.00) (*p* = 0.43) (1.9)

95% CI, 95% Confidence Intervals; US-CNB, Ultrasound-guided Core Needle Biopsy.

### Postoperative complications

The authors found that postoperative complications of US-CNB occurred in 12 of all studies. Of these, postoperative complications occurred in 65 of 2521 patients. In addition, the authors introduced data on postoperative complications of US-FNA for comparison with US-CNB. After comprehensive data analysis, the authors found that US-FNA was associated with fewer postoperative complications than US-CNB. The authors also found that US-FNA was lower than US-CNB in terms of patient pain perception.^28^

## Discussion

The presence of ALNs metastases is an important indicator of poor prognosis in BC patients. Accurate preoperative staging of ALNs in BC allows early assessment of the patient's axillary status, development of treatment strategies, and assessment of prognosis for individualized patient treatment.^31^ Two large randomized trials of ALNs, ACOSOG's-Z0011^2^ and IBCSG-23-01^32^ change conventional axillary therapy to reduce unnecessary ALND and improve patients' postoperative quality of life. In the future, axillary management may become more precise and personalized, and accurate preoperative staging of the axilla may reduce the need for additional surgery, further reducing the use of ALND. Imaging is often used for preoperative screening and assessment of patients. In general, the devices used to image the ALNs include nuclear medicine, magnetic resonance, and ultrasound, of which ultrasound is the most commonly used and can easily obtain high-quality images of the ALNs under non-invasive conditions.^33^ With the development of ultrasound technology, the use of US puncture biopsy techniques has become more widespread, of which US-CNB has a low False Negative Rate (FNR) and has been shown to have excellent safety and accuracy.^20^

By summarising all the studies, the authors found the following advantages of US-CNB in the preoperative detection of ALNs metastases in BC patients, including 1) No general anesthesia is required; 2) Save time and prevent a second procedure or unnecessary SLNB, precise axilla staging can be obtained at the first visit; 3) Better diagnostic performance than FNA; 4) Ability to run immunohistological tests on extracted samples; and 5) An effective and safe diagnostic tool.^34^

In recent years, a growing body of literature has demonstrated that diagnostic results are more accurate when the two types of detection are used in combination.^35^ Cserni and colleagues showed that combining the two assays for diagnostic purposes resulted in higher specificity and sensitivity, as well as fewer false negatives, thus improving the performance of the assay and providing maximum diagnostic accuracy for patients.^36^ The combination of dye-isotope and US-CNB can help identify some lymph nodes that are not visible on ultrasound.^19^ Therefore, US-CNB can be used in combination with other methods to improve diagnostic accuracy.

The number of punctures of a biopsy may have an impact on the accuracy of the diagnosis. The study by Macaskill and colleagues showed that the diagnostic rate increased with the number of punctures, in their trial the rate was 81.8% (45/55) for the first puncture, 96.4% (53/55) for the second, and 100% (55/55) for the third.^37^ In the above study, the diagnostic rate of the second puncture was already high. In addition, the continued use of puncture to obtain additional samples may improve the diagnostic rate of some patients with small ALNs involvement.^17^

The most critical aspect of US-CNB operation is avoiding damage to adjacent structures, such as blood vessels or nerves, and the vast majority of anterior lymph nodes are located caudal to the axilla. For ALNs with a complex surrounding structure, a modified location and method of puncture can safely access a greater proportion of the ALNs and effectively reduce complications.^38^ However, part of the lymph node is located higher up in the armpit, close to the major vessels, and the use of US-CNB poses a safety risk for these patients.^27^ In rare situations where the anterior axillary lymph nodes are located near large blood vessels or deep in the thoracic wall, the safer method instead of trying US-CNB would be the one to choose. Therefore, a good understanding of the structure of ALNs can increase the safety of using US-CNBs and reduce complications.

The authors found that a small number of patients developed some post-operative complications, including pain, hematoma, bleeding, bruising, and pneumothorax. Of these, pain was the most common postoperative complication. To date, no serious postoperative complications have been reported in the literature. During the process of puncture, the tumor may undergo malignant seeding along the trajectory of the puncture, or minor lesions may occur around the tumor and result in incomplete resection of the tumor, which may lead to tumor recurrence.^39^ In some cases, US-CNB may cause a hematoma or fibrosis after the puncture, making it difficult to accurately identify the location and shape of lymph nodes during surgery.^40^ Different approaches, patient choice, and operator proficiency may influence the development of complications.^41^ Continuous ultrasound monitoring, appropriate needle selection, improved operator proficiency, and appropriate patient selection can effectively prevent potential complications of US-CNB.^25^

In terms of needle selection, it has been previously reported in the literature that minor complications occurred in only a small proportion of patients in previous studies using conventional automated needles for puncture. However, in the study by Abdsaleh and colleagues, a modified type of needle was used, and patients had no postoperative complications.^42^

To date, it has been debated whether US-CNB is superior to US-FNA in detecting ALNs in BC patients. Among the studies the authors included, eight studies performed comparative analyses between US-CNB and US-FNA. The authors found that the sensitivity of US-CNB was better than that of US-FNA, but both were similar in terms of specificity. Previous studies have shown that the inadequate rate of US-FNA samples ranged from 0% to 54%.^43^ The higher specificity of US-FNA in our study may be associated with the choice of trials with bigger tumors or the exclusion of patients with insufficient samples.^44^ US-CNB uses a larger needle size than US-FNA for sample collection and may obtain more samples. For the identification of micro-metastases, the ability of US-CNB to identify micro-metastases in lymph nodes is superior to that of US-FNA, and US-CNB can measure the size of metastatic deposits to identify micro-metastases in ALNs.^21^ the ability of US-CNB to visualize the structure of the lymph nodes can be of significant help in the diagnosis of pathology.^45^ US-CNB shows ALNs structures that can be evaluated by anatomic pathologists, whereas US-FNA only shows cells that need to be evaluated by cytopathologists, and the experience of the pathologist may also influence the differences in the reported procedures.^46^ In addition, samples obtained by US-CNB contain sufficient RNA and DNA to allow molecular experiments and immunohistochemistry to further assess the status of tumor biomarkers and provide a useful basis for clinical treatment planning. Therefore, US-CNB is superior to US-FNA in the diagnosis of ALNs in BC patients.

A number of meta-analyses have previously compared US-CNB with US-FNA. A meta-analysis by Hous-Sami and colleagues found no significant difference between the two, and the lack of a large enough sample to include in the study made the study limited and the accuracy of the results limited.^47^ Balasubramanian and colleagues showed that US-CNB is a superior diagnostic technique to US-FNA for ALNs metastasis of BC was detected, and the sensitivity of US-CNB was 0.88 (95% CI 0.84‒0.91; *p* = 0.12) vs. 0.73 (95% CI 0.69‒0.76; *p* = 0.91) for US-FNA, the specificity was similar and the AUC were 0.984 and 0.979.^48^ In addition to that, Pyo and colleagues investigated the accuracy of the two methods according to the cytological preparation method and found that there was no significant difference between the different cytological preparation methods of US-FNA and the sensitivity of US-CNB was higher than that of US-FNA.^49^

US-CNB may be lower than US-FNA in terms of FNR.^20^ In addition, a recent study found that FNR may be strongly associated with the size of suspicious ALNs in BC patients.^50^ In the majority of cases, false negatives occur may be because the ALNs cannot be palpated in the fatty tissue of the axilla or because of the inability to identify lymph nodes on ultrasound when they occur during inflammatory disease. As there are differences between the different pathological types of BC, there will be some false negatives due to different types. [51,52].

The authors found a moderate degree of heterogeneity in the overall sensitivity of the US-CNB. Firstly, the use of NAC treatment before surgery may alter the initial condition of the axilla, which may lead to false negative results and then affect the accuracy of US-CNB. Secondly, there are differences between regions and these differences can lead to the creation of heterogeneity. Thirdly, different sizes and grades of BC masses, with different probabilities of metastasis to ALNs, may have an impact on the accuracy of the US-CNB. As the BC masses grade and size increased, the risk of ipsilateral ALNs metastasis increased, as did the sensitivity of US-CNB.^26^ Finally, it is also possible that the number of punctures may have an impact on the accuracy of the US-CNB, as sometimes micrometastases may be present in the ALNs of BC patients who require multiple punctures to be diagnosed.

The authors strictly followed the systematic review method during the research process, the included literature was of high quality, and a large number of clinical samples were included, which gives our results good credibility.

However, there are several limitations to our meta-analysis that need to be considered. First of all, there was a moderate degree of heterogeneity in the overall sensitivity of US-CNB in our analyses, and the authors only performed subgroup analyses for four of these factors; the remaining factors were not included. Other factors may also influence the accuracy of the US-CNB, such as the ethnicity of the patient, the timing of the US-CNB assessment, and the degree of illness. Secondly, there is insufficient information in some of the literature to obtain sufficient information, which may lead to some bias in the results. Thirdly, the different equipment used in each study and the different skills of the operators made it impossible to achieve a uniform standard, which may have affected the credibility of the results to some extent. However, the authors did not analyze these factors and could not eliminate their influence on the results. Fourthly, some of the literature the authors included was from small sample studies, which may have influenced the results. Finally, the authors only selected articles published in English and did not select articles published in other languages, which biased our literature search.

Although our meta-analysis has many limitations, the results of our study can provide great help in the preoperative evaluation of ALNs metastasis in BC patients. The preoperative use of US-CNB can help patients significantly reduce the time and cost of diagnosis and reduce the pain of secondary surgery. However, due to the lack of data, more large studies are needed to further confirm US-CNB as a screening criterion and diagnostic tool for ALNs metastasis in BC patients.

## Conclusions

In conclusion, US-CNB has good specificity, sensitivity, and accuracy in the diagnosis of ALNs metastases in BC patients and can be used as a preoperative detection method to facilitate early axillary staging and enable the personalized treatment plan for patients.

## Authors’ contributions

Feng L had full access to all the data in the study and take responsibility for the integrity of the data and the accuracy of the data analysis. Concept and design: Feng L, Qi X, Jiale W. Acquisition, analysis, or interpretation of data: Feng L, Qi X, Jiale W, Jing W. Drafting of the manuscript: Feng L, Qi X, Jiale W. Critical revision of the manuscript for important intellectualcontent: Feng L . Statistical analysis: Qian Y, Jing W, Runzhao G. Administrative, technical, or material support: Qian Y, Jing W, Runzhao G. Supervision: Jing W. All authors have read and approved the manuscript.

## Conflicts of interest

The authors declare that the research was carried out without any commercial or financial ties that could have created a potential conflict of interest.
